# Atoxyl in Syphilis

**Published:** 1908-11-07

**Authors:** 


### THERAPEUTICS.

### ATOXYL IN SYPHILIS.

THE improvement brought about in cases of the dread malady sleeping sickness by atoxyl treatment, and the experiments that have recently been carried out to determine why atoxyl needs assistance from some other drug such as perchloride of mercury if the best results are to be obtained, have led to the use of atoxyl—sodium anilarsenate—in the treatment of other lesions in which mercury or arsenic or both do good, notably in syphilis. Fortunately it is not often nowadays that more than the ordinary treatment for syphilis is required. Occasionally, however, one of those extreme and resistant cases that used not to be uncommon is met with. It is in these severe cases in which all the ordinary measures have been conscientiously tried, but without complete success, that something more than the ordinary is needed; and it is here that atoxyl promises to be very useful. Chirivino records four cases of extremely bad tertiary syphilis treated and either cured or very much relieved by atoxyl when mercurials and iodides had not succeeded. His paper is in the "Riform. Med." for 1907. Gummatous deposits and lesions of the bones were completely cured. It is probable that the use of atoxyl by itself would be of little benefit compared to the administration of this drug in conjunction with, or soon after, iodides and mercury. In some cases there is intolerance of the remedy even in small doses, but as an ordinary dose Chirivino recommends a grain and a half hypodermically once a day. He does not increase the dose in the method followed in sleeping sickness. He very much prefers the small dose, and never exceeds three grains per diem. The drug is made up in a specially sterilised 10 per cent. solution, under the name of portokil, which is ready for injection. When intolerance exists, it soon shows itself, and the drug must be stopped. In other cases, no untoward symptoms occur, appetite and general nutrition are reported to improve rapidly, and the tertiary lesions begin to heal almost at once.

As an improvement even upon atoxyl, Colonel F. J. Lambkin, R.A.M.C., has quite recently recommended an allied substance containing, like atoxyl, a very large percentage of arsenic in organic combination. This is sodium para-amino-phenyl-arsenate of which the probable formula is

$$C_6H_4.NH_2.AsO.OH.ONa,5H_2O.$$

It contains 22.8 per cent. of arsenic, and yet is far less toxic than is liquor arsenicalis itself. It may be employed without ill effects in patients who cannot tolerate atoxyl; and apparently the results obtained from this compound—known as soamin—are better than those which follow the giving of atoxyl even in cases where the latter might be well tolerated. Administration is by hypodermic or by intramuscular injection as in the case of atoxyl. Mercurial treatment is required too, but it has to be carried on alternately with and not simultaneously with the soamin injections. Colonel Lambkin's directions appear to be that before beginning mercurial treatment, or *vice versa*, it is well to wait fifteen days. The results in syphilitic cases have been such as to warrant the soamin being called a second specific for syphilis.

